# Analysis of correlated mutations in Ras G-domain

**DOI:** 10.6026/97320630013174

**Published:** 2017-06-30

**Authors:** Ekta Pathak

**Affiliations:** 1 Bioinformatics Department, MMV, Banaras Hindu University

**Keywords:** Ras, GTPases, G-domain, Coevolution, Correlated Mutation

## Abstract

Ras GTPases are most prevalent proto-oncogenes in human cancer. Mutations in Ras remain untreatable more than three decades after the initial discovery. At the amino acid level, some residues under physical or functional constraints exhibit correlated mutations also known as coevolving/covariant residues. Revealing intra-molecular co-evolution between amino acid sites of proteins has become an emerging area of research as it enlightens the importance of variable regions. Here, I have identified and analyzed the coevolving residues in the Ras GTP binding domain (G-domain). The obtained covariant residue position data correlate well with the known experimental data on functionally important residues. Therefore, it is of interest to understand these residue co-variations for designing protein engineering experiments and target oncogenic Ras GTPases.

## Background

Ras GTPases are most prevalent proto-oncogenes in human cancer [1]. Mutations in Ras remain untreatable more than three decades after the initial discovery. The Ras GTP binding domain (G-domain) functions as molecular switches regulating pathways responsible for proliferation and cell survival [2]. An effector molecule, GTPases-activating proteins (GAPs) stimulate the hydrolysis of GTP to GDP to switch off signaling. GDP dissociation and GTP binding is regulated by Guanine nucleotide exchange factors (GEFs) [3, 4]. The binding and hydrolysis of guanine nucleotides cause significant conformational changes in two functional loop regions, Switch I (residue position 25-40) and Switch II (residue position: 57-75) that surround the phosphate group of the nucleotide. The stabilization of the enzymesubstrate complex and the hydrolysis of GTP are facilitated by set of five conserved motifs. G1 (G/AXXXXGKS/T) motif or the Ploop interacts with α- and β-phosphate groups. A conserved Thr35 residue of G2 (XTX) motif is part of switch I. Thr35 of Ras interacts with Mg2+ and essential for GTP hydrolysis [5]. G2 motif is also called the effectors loop because it is a site for effector and GAP binding. The G3 (DXXG) motif is part of switch II region and provides residues for Mg2+ binding and γ-phosphate binding. G4 or N/TKXD motif and G5 or the SAK/L motifs help in guanine recognition [6].

Hidden information about protein structure and function can be extracted by looking at the correlated mutational behavior of the amino acid residues positions [7]. An amino acid substitution, which partly destabilizes the protein structure or function, could be corrected by a substitution at different site [8]. This change in amino acid relative to another is known as correlated mutation or co-evolution or co variation. These residues at some sites strongly affect the evolution of certain other sites in the three dimensional structure of the protein. Residue co-evolution allows protein to maintain its overall structural-functional integrity while enabling it to acquire specific functional modifications [7, 8].

Here, coevolving residue positions were identified and mapped onto the Ras G-domain. These residue positions were calculated using multiple sequence alignment of Ras superfamily (Ras, Rab, Ran, Rho and Arf) members. A comprehensive literature survey using PubMed and PubMed central search was performed to retrieve the description of the experimentally verified functional information for the predicted covariant residue positions. The presented correlated mutation data will be of interest to the wet lab experimentalist to unlock the secret behind the action of undruggable Ras.

## Methodology

### Retrieval of Ras G-domain data

RAS protein sequence was retrieved from UniProt database (UniProtKB accession number - P78460_HUMAN). PSI-Blast search tool of NCBI was used to identify the homologous sequences of Ras GTPases. The results of PSI-blast search were manually screened to include Ras superfamily members such as Rab, Ran, Rho and so on. These sequences were aligned using ClustalX 2.1 tool (http://www.clustal.org/clustal2/). For analysis, only the GTP binding domains that had conserved G1, G2, G3 and G4 motifs were retained, after deleting neighboring domains at their N and/or C terminal sides. For structural study, experimental structure of Ras (PDBID: 5P21) was retrieved from the Protein Data Bank (http://www.rcsb.org/pdb).

### Prediction of coevolving sites

In order to predict the coevolving sites, the MSA file containing the G-domain sequence of Ras superfamily were submitted to InterMap3D server (http://www.cbs.dtu.dk/services/InterMap3D/). InterMap3D predicts co-evolving pairs of amino acids from an alignment of protein sequence [9]. Here, "RCW MI" method was chosen to predict the coevolving positions. The identified Coevolutionary positions were mapped onto the three dimensional structure of the Ras G-domain (PDBID: 5P21) using UCSF Chimera (https://www.cgl.ucsf.edu/chimera/). PubMed and PMC search was performed to retrieve the description of the experimentally verified functional information for predicted covariant residue positions.

## Results and Discussion

Usually, conserved residue positions are used to identify the functionally important sites in proteins, and a little attention has been given to the residues other than the conserved ones. At the primary structure level, some amino acid residues under physical or functional constraints exhibit correlated mutations or coevolution. Revealing intra-molecular Coevolutionary site has become an emerging area of research as it enlightens the importance of variable regions [7, 8].

**Figure 1:**
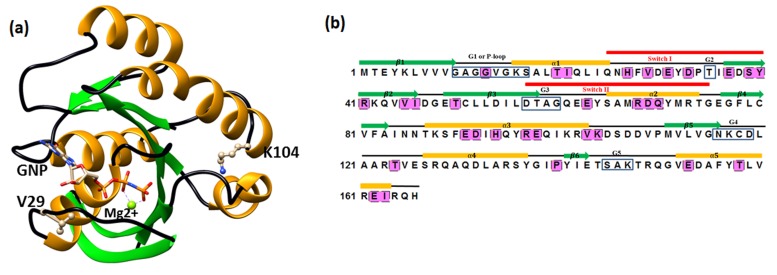
A distribution map of Coevolving residue positions onto the Ras GTPase. A) Ras G-domain (PDBID: 5P21) showing the correlated positions V29 and K104; these two positions showed a high frequency (eight and seven, respectively) of correlated mutations with other residue positions. B) Coevolutionary sites are shown in shaded pink color. G1-G5 motifs are marked with rectangle. Switch I and Switch II regions are shown in red bar.

The coevolving residues in the Ras GTP binding domain were identified and analyzed using InterMap3D (see methods). Mapped onto Ras G-domain; PDBID: 5P21 (Figure 1). A list of 35 pairs of coevolving residue pairs was identified (Table 1). Analysis of covariant residue revealed that Switch I (residue position 25-40) and Switch II (residue position 57-75) of Gdomain harbor seven (H27, V29, E31, D33, E37, S39, and Y40) and four covariant residues (E63, R68, D69, Q70), respectively. The coevolving residues G13, V29, E31 and D33 were observed within 5Å from the ligand GNP (Figure 1 and Table 2). Noticeably, G13 from p-loop coevolved with V29 of Switch I and a set of four pairs of residues (V29-D69, V29-Q70, S39-E63 and S39-R68) from Switch I-Switch II regions showed co-evolution with each other. The conformational changes at switches depict the active and inactive state of the Ras signaling process [6]. Switch I facilitates GTP hydrolysis through GAP molecules whereas Switch II selectively binds GEFs to carry out exchange of GTP and GDP [3, 4]. Therefore, presence of coevolving residue positions around the catalytic pocket indicates their role in imparting functional diversity. However, a larger number of covariant site at Switch I, with seven covariant sites, compared to Switch II with four covariant sites suggest a high vulnerability and a larger role of switch I compared to switch II in regulation of GTPase cycle and cellular signaling. It will be of interest to explore the role of correlated amino acid residues, which are located away (more than 5Å) from the GTP binding sites (Table 2). These residues are: T20, I21, R41, V45, I46, T50, E92, D93, H95, R98, E99, V103, K104, T124, P140, E153, T158, E162, and I163 (Figure 1 and Table 2).

In order to understand, verify and scrutinize the specific molecular and functional role of the reported covariant positions, a comprehensive literature search was performed. PubMed and PMC search revealed that most of the identified covariant residues were associated with regulation of function of Ras (Table 3). As shown in Table 3, implications of covariant residues of G-domain were found reported in regulation of GTPase cycle, effector binding to Switch I and Switch II region, mediation of water molecule in hydrolysis and sharing regions of allosteric sites [10-22]. Intriguingly, V29 position of Switch I and K104, located away from the pocket, (Figure 1A) showed Coevolutionary pattern with eight and seven other residue positions, respectively (Table 1 and Table 3). Although, analysis of Ras (PDBID: 5P21) revealed that V29 interacts with sugar moiety of GNP (a GTP analog) (Figure 1A), and it is also known to coordinate with conserved water in the catalytic pocket which is essential for hydrolase activity of GTPases [12]. Also, modification at K104 by acetylation affects the conformational stability of the Switch II domain, which is critical for the ability of RAS to interact with guanine nucleotide exchange factors [20]. However, Coevolutionary pressure of eight and seven coevolving residue pairs associated with V29 and K104, respectively, indicate a larger role to be played by these positions, and hence opens up a question for future investigation.

Here, covariant residues T20, H27, E31, I46, T50, Q70, E91, D92, E98, V103, T124, P140, T158 and E162 were identified as novel sites (Table 3). No experimental report was available, to the best of my knowledge, for these sites. Therefore, it is of interest to understand the role of these residue positions in the functionality of Ras Superfamily and to target oncogenic Ras.

## Conclusion

Coevolving residue positions are functionally important sites and point mutations at these sites result in conformational change in Ras. Here, residues T20, H27, E31, I46, T50, Q70, E91, D92, E98, V103, T124, P140, T158 and E162 were identified as novel covariant sites for which functional implications are yet to be discovered. Also, understanding the role of co-variant residues with high frequency of correlated mutation pairs, such as V29 and K104, might open new avenues in designing experiments to target Ras oncogene.

## Figures and Tables

**Table 1:** List of 35 coevolving amino acid residue pairs in Ras G-domain

Amino Acid	Residue Position 1	Amino Acid	Residue Position 2
G	13	V	29
T	20	D	92
I	21	V	29
H	27	E	98
V	29	D	33
V	29	S	39
V	29	Q	70
V	29	D	69
V	29	K	104
V	29	T	158
E	31	V	45
D	33	R	41
D	33	K	104
D	33	E	153
E	37	K	104
S	39	R	68
S	39	D	92
S	39	T	158
Y	40	E	63
R	41	K	104
V	45	E	162
I	46	T	124
T	50	P	140
R	68	Q	70
D	69	V	103
D	69	K	104
D	69	D	92
D	69	E	153
E	91	E	162
D	92	K	104
H	94	I	163
R	97	E	98
R	97	V	103
V	103	K	104
E	153	E	162

**Table 2:** A map of coevolving residues onto the Ras G-domain

G-DOMAIN	Coevolving Residue Positions shown on Ras (PDBID: 5P21)
Switch I (Effectors binding site)	H27, V29, E31, D33, E37, S39, Y40
Switch II (Effectors binding site)	E63, R68, D69, Q70
Nucleotide (GDP/GTP) binding site (Within 5Å of nucleotide)	G13, V29, E31, D33
Regions away from ligand binding site (>5Å of nucleotide)	T20, I21, R41, V45, I46, T50, E92, D93, H95, R98 E99, V103, K104, T124, P140, E153, T158, E162, I163

**Table 3:** Coevolving residue positions and their known functional implications

Coevolving residue positions	Degree of Co-Variation	Functions (Indicated in literature)	References (Literature Survey)
G13	1	Impairs GTP/GDP cycle	[10]
T20	1	Not known	NA
I21	1	Plays a crucial role in Switch I transition	[11]
H27	1	Not known	NA
V29	8	Coordinates conserved water molecule	[12]
E31	1	Not known	NA
D33	4	Required for interaction with GAP	[13]
E37	1	Located at PI3K/Ras interface	[14]
S39	4	Located at allosteric site	[11]
Y40	1	Located at PI3K/Ras interface	[14]
R41	2	Important for SOS-catalyzed nucleotide exchange on Ras	[15]
V45	2	Required for interaction with GAP	[16]
I46	1	Not known	NA
T50	1	Not known	NA
E 63	1	Important for CDC25GEF-mediated conformational changes that decreases affinity for GDP and increases affinity for GTP	[17], [18]
R68	2	Contributes to order the switch II region and coordinates a water molecule in active site	[12],[13]
D69	5	Important for CDC25GEF-mediated conformational changes that decreases affinity for GDP and increases affinity for GTP	[18]
Q70	2	Not known	NA
E91	1	Not known	NA
D92	4	Not known	NA
H94	1	Found at allosteric switch	[19]
R97	2	R97G found in human tumor; found at allosteric switch	[19]
E98	2	Not known	NA
V103	3	Not known	NA
K104	7	Acetylation of K104 affects the efficiency of nucleotide exchange and also suppresses the oncogenic activity RAS.	[20]
T124	1	Not known	NA
P140	1	Not known	NA
E153	3	Important for interaction with the membrane.	[21]
T158	2	Not known	NA
E162	3	Not known	NA
I163	1	Located at allosteric site	[22]
